# Measles a Dangerous Disease

**Published:** 1896-05

**Authors:** 


### MEASLES A DANGEROUS DISEASE.

There is quite a prevalent but erroneous idea that measles is not a dangerous disease, and hence but little effort is made to restrict its spread in most localities. However, in England, and some American States, statistics have shown the magnitude of the danger resulting from this opinion and neglect, and also the great benefit following the application to this disease of the preventive measures generally used against other communicable diseases.

The Michigan State Board of Health has taken the lead in this important field of State preventive medicine. This it is enabled to do, because for years that State has enforced a thorough system of gathering vital statistics, which is placed at the disposal of its Health Board; which also is furnished with a large clerical force, and means of communicating with every neighborhood in the State, and of insuring compliance with its rules and regulations.

The Michigan State Board of Health classes measles with "diseases dangerous to the public health," and which must be restricted under the law; because it is a communicable disease, and, therefore, preventable; because it occasions a large number of deaths each year in Michigan, and causes several thousands of cases of sickness each year. Also it frequently injures or destroys the organs of sight or hearing. It prepares the way for, and is closely followed by, pneumonia and consumption. In England, where it is very prevalent, it destroys more lives than diphtheria and scarlet fever combined.

By reference to the voluminous and elaborate reports of the Michigan State Board of Health for thirteen years, inclusive, it will be seen that great labor has been bestowed upon the prevention of measles, and with marked beneficial results. In the latest (or twenty-third annual report), attention is called to two erroneous and very harmful beliefs, to wit: That measles cannot ultimately be escaped any more than teething, and that the least dangerous time for persons to have the disease is while quite young children. The carefully studied statistics of Michigan demonstrate that measles is a preventable disease, and that it is more fatal to children under ten years of age than to older persons.

Doubtless, if Tennessee had such a system for collecting vital statistics as Michigan has, and if like study was bestowed upon the hitherto underrated disease, measles, the same fact would be apparent here. Beyond question the medical profession in Tennessee, and many other localities, has been too careless in this regard, and should hereafter profit by the long and patient efforts of Dr. H. B. Baker to wake us up.—*State Board of Health Bulletin of Tennessee*, March 20th, 1896.

